# Bench-Scale Evaluation of Hydraulic Performance and Rejection of Bisphenol-A and Estradiol by Different Nanofiltration Membranes as a Post-Treatment Step at the Lago Norte WTP—Brasília/DF, Brazil

**DOI:** 10.3390/membranes16070242

**Published:** 2026-07-17

**Authors:** Bianca Campos Gonçalves, Cristina Celia Silveira Brandão, Sara Regina Morais Kollar

**Affiliations:** Environmental Technology and Water Resources Post-Graduation Program, Department of Civil and Environmental Engineering, University of Brasília (UnB), Brasília 70910-900, Brazil; 231119312@aluno.unb.br (B.C.G.);

**Keywords:** water treatment, nanofiltration, bisphenol-a, estradiol, endocrine disruptors

## Abstract

Emerging micropollutants in drinking water sources represents a growing challenge for water treatment systems. Bisphenol-A (BPA) and 17β-estradiol (E2) are endocrine disruptors widely detected in aquatic matrices that are not efficiently removed by conventional treatment. This study evaluated, at the bench scale, operational performance and rejection of BPA and E2 by three nanofiltration membranes—NFM1, NFM2 and NFM3—operating at 8 bar and using ultrafiltered water from the Lago Norte Water Treatment Plant (WTP), Brasília/DF, Brazil, spiked with both compounds at 150–250 µg/L, as the feed matrix. Hydraulic parameters, such as permeate flux and water permeability, were assessed alongside rejection. NFM1 exhibited the highest permeate fluxes (136.1 and 171.7 L/h·m^2^); however, it showed the lowest rejection (E2: 57–73%; BPA: 28–60%). The NFM2 membrane showed intermediate rejection behavior (E2: 86–89%; BPA: 67–91%) but presented the lowest permeate flux (51.2 to 63.3 L/h·m^2^). The NFM3 membrane presented the highest rejection and greatest operational stability (E2: 90–95%; BPA: 95–97%), with a permeate flux of 59.1 to 67.0 L/h·m^2^. Size exclusion was the predominant removal mechanism, though adsorption also contributed during the initial hours of operation. The results confirm a trade-off between permeate production and contaminant rejection, with no single membrane outperforming all others across all criteria.

## 1. Introduction

The occurrence of contaminants of emerging concern in aquatic environments has become one of the main challenges for drinking water treatment systems. These substances are potentially toxic to ecosystems and human health and, in most cases, lack regulatory drinking water standards. Among these contaminants, endocrine-disrupting compounds (EDCs) stand out due to their ability to interfere with the hormonal system of aquatic living organisms and humans, even at trace concentrations around ng/L and µg/L [1].

Within EDCs, Bisphenol-A (BPA) and 17β-estradiol (E2) stand out because of their widespread occurrence and endocrine activity. BPA is extensively used in the production of polycarbonate plastics and epoxy resins, whereas E2 is a natural estrogen hormone continuously released into aquatic environments, mainly through domestic wastewater discharges.

Several studies have reported the detection of BPA and E2 in surface waters, wastewater effluents, and even treated drinking water [2,3,4,5,6,7,8,9]. Their persistence in aquatic environments and in treated water indicates that conventional water treatment technologies cannot efficiently remove these micropollutants. Advanced treatment technologies, such as membrane separation processes, have emerged as promising alternatives for the removal of these EDCs, especially nanofiltration (NF) membranes [10,11,12,13,14].

Although several studies have evaluated endocrine disruptor rejection by NF membranes, many investigations were performed using synthetic feed solutions or deionized water. Therefore, studies using real water as a matrix are still necessary to better understand membrane behavior under more realistic operating conditions.

In addition, the rejection of BPA and E2 by NF membranes depends on several factors, including membrane characteristics, such as molecular weight cut-off (MWCO), pore size, and hydrophobicity; operating conditions, like pressure, pH, and temperature; feed water composition; and physicochemical properties of target compounds [10,15,16].

In this context, the present study evaluated the hydraulic performance (permeate flux, transmembrane pressure (TMP), and hydraulic permeability) and the rejection of BPA and E2 by three different nanofiltration membranes, using ultrafiltered water produced at the Lago Norte Water Treatment Plant in Brasília, Brazil, as a matrix, in order to evaluate the use of nanofiltration as a post-treatment step at the Lago Norte WTP.

## 2. Materials and Methods

### 2.1. Water Matrix

The Paranoá Lake Water Supply System (Brasília, Brazil) includes both the raw water intake structure, located at the Ribeirão do Torto branch of Paranoá Lake, and the treatment plant itself, which supplies several regions of Brasília and the surrounding area.

The Lago Norte WTP operates using ultrafiltration (UF) membrane technology with hollow-fiber membrane modules. In total, there are 630 membrane modules, each with an effective filtration area of 77 m^2^, resulting in a total filtration area of 48,510 m^2^ and a production capacity of up to 700 L/s.

Both the water collected from Paranoá Lake and the treated water produced at the Lago Norte Water Treatment Plant showed low physicochemical variability throughout the year. In addition, the low hardness, turbidity, and natural organic matter (NOM) values observed in the ultrafiltered water used as the feed matrix in the present study indicate its high water quality [15]. The treated water typically presents turbidity values around 0.08 NTU [17].

Table 1 presents the physicochemical characteristics of the ultrafiltered water used as the feed matrix. The results indicate low concentrations of dissolved species and suspended matter, reflecting the good quality of the feed water. The low turbidity (0.27–0.44 NTU) and absorbance (0.004–0.008) values indicate a low content of suspended particles and UV-absorbing organic matter. Likewise, the low alkalinity, conductivity, hardness, and calcium and magnesium concentrations demonstrate the low mineralization of the feed water. Overall, these characteristics suggest that the feed matrix is unlikely to significantly influence the nanofiltration separation mechanisms, allowing the observed membrane performance to be primarily attributed to the interactions between the membranes and target contaminants.

For the present study, ultrafiltered water used as a matrix was collected directly from the permeate storage tank used for membrane backwashing. This sampling point was selected because it is located before sodium hypochlorite addition, avoiding potential interference of chlorine with the nanofiltration membrane performance during the experiments. Depending on the number of experiments performed, between 40 and 80 L of ultrafiltered water were collected weekly using 10 L glass containers to minimize contamination.

### 2.2. Contaminants and Feed Water Preparation

The feed water consisted of ultrafiltrated water from the Lago Norte WTP, spiked with both BPA and E2.

Stock solutions of each contaminant were prepared separately in HPLC-grade methanol (Sigma-Aldrich, São Paulo, SP, Brazil) at a concentration of 100 mg/L. The solutions were stored in amber flasks under refrigeration for up to one month. The stock solutions were used both for preparation of the feed solution and for calibration standards employed in the analytical quantification of BPA and E2 (Sigma-Aldrich, São Paulo, SP, Brazil).

The concentrations of BPA and E2 in the feed water ranged from 150 to 250 µg/L. These concentrations were based on previous studies [11,12,13], as well as on the detection and quantification limits of the analytical method. This concentration range allowed the analyte concentrations to be quantified without the need for concentration step of E2 and BPA in the permeate samples, as the permeate volumes produced during the bench-scale experiments were insufficient to support such procedures.

BPA and E2 were purchased from Sigma-Aldrich (São Paulo, Brazil), both with purity levels higher than 99% and of HPLC grade. The physicochemical properties of BPA and E2 are presented in Table 2.

### 2.3. Nanofiltration Membranes

Membranes NFM1 and NFM3 were selected because they are well-established membranes commonly used for micropollutant removal in drinking water treatment [11,12,13,14,18,19,21]. In contrast, NFM2 was selected because it exhibits characteristics that are intermediate between those of the other two membranes, allowing the evaluation of a broader range of membrane characteristics.

The main characteristics of the three NF membranes evaluated are shown in Table 3.

NFM3 and NFM1 are thin-film composite membranes comprising a thin polyamide active layer over a microporous polysulfone support layer. However, NFM1 contains a semi-aromatic polypiperazine-amide active layer, whereas NFM3 presents a fully aromatic polyamide structure [18]. NFM3 is considered a tight membrane, with smaller pore sizes and lower hydraulic permeability, while NFM1 is classified as a more “open” membrane, exhibiting larger pores and higher permeability.

The NFM2 membrane is also a thin-film composite membrane, consisting of a polypiperazine-amide active layer supported by non-woven polyester. According to the manufacturer, this configuration provides a dense and mechanically resistant structure. Its molecular weight cut-off (200–300 Da) and average pore diameter indicate structural similarity to NFM1 regarding size exclusion behavior. However, its lower hydraulic permeability suggests a more restrictive structure to water transport, possibly associated with differences in active layer thickness or compaction. On the other hand, the membrane supplier reports an MWCO of 200 Da for NFM2, which, together with its water permeability, indicates that its separation behavior may be closer to that of the NFM3 membrane.

Based solely on the molecular weight cut-off (MWCO), the NFM3 membrane would be expected to exhibit higher rejection. Furthermore, E2 would be expected to show higher rejection than BPA, as its molecular weight (272 g mol^−1^) is greater than that of BPA (228 g mol^−1^).

Although classification criteria vary in the literature, membrane hydrophobicity is commonly evaluated through contact angle measurements. In general, higher contact angles indicate more hydrophobic surfaces. Surface roughness also influences contact angle measurements and membrane wettability [29,30,31]. Based on reported contact angle values, NFM3 (42.2°) presents intermediate behavior, while NFM1 (23.4°) and NFM2 (28°) are predominantly hydrophilic.

The authors suggested that the adsorption mechanism in nanofiltration membranes is governed by hydrophobic interactions between the contaminants and the membrane surface, which becomes more favorable as membrane hydrophobicity increases. Accordingly, the higher hydrophobicity of NFM3 may also contribute to a higher rejection of BPA and E2 in comparison to NFM1 and NFM2.

Flat-sheet membrane samples (from now on referred to as membrane sheets) were acquired from Sterlitech Corporation (Auburn, AL, USA). Each membrane sheet measures 19.1 × 14 cm, with a usable area of 140 cm^2^.

### 2.4. Experimental Procedure

**Figure 1:**
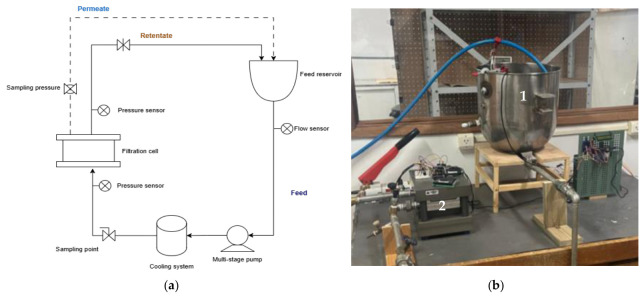
(**a**) Nanofiltration system diagram. (**b**) Partial view of nanofiltration system: (1) feed reservoir with capacity of 20 L; (2) filtration cell (Sepa CF Cell—Sterlitech, Auburn, AL, USA), with an effective filtration area of 140 cm^2^.

As shown in Figure 1, the experiments were carried out in a bench-scale nanofiltration system (Sepa CF Cell – Sterlitech, USA), operating in crossflow mode under a pressure of 8 bar. The retentate was continuously recirculated to the feed tank, while permeate recirculation occurred intermittently due to periodic sample collection for analysis.

The nanofiltration system also included pressure, flow, and temperature sensors, as well as a manual pump that was used to keep the nanofiltration cell pressurized and prevent its opening during system operation. The pressure sensors model G1/4 1.2 MPa (Benser, Nanjing, China ) operated within a voltage range of 0.5–4.5 V, supported temperatures between 20 and 105 °C, and had a maximum operating pressure of 2.4 MPa (24 bar). The flow sensor model YF-S201(Bolsen, Foshan, China ) operated within a voltage range of 5–24 V and a flow range between 1 and 30 L/min. These sensors were managed by a microcontroller on the Arduino platform.

The permeate flow rate was determined by the volumetric method, since its values were below the sensor detection limit, around 1 L/min. For this purpose, a stopwatch and a 25 mL glass graduated cylinder were used. The concentrate flow rate was calculated from the difference between the feed and permeate flow rates.

Prior to system operation, each membrane sheet was soaked in ultrapure water for at least 24 h, so that it could hydrate while removing surface impurities. The experimental system was cleaned by recirculating ultrapure water. Then, the membrane was compacted by recirculating ultrapure water pressurized at 8.5–9 bar for 30 min.

Prior to experiments with BPA and E2, permeability tests were performed using ultrapure water. Initially, the operating pressure was adjusted to 2 bar and, after flux stabilization, permeate flow rate was measured by the volumetric method, while feed flow rate and concentrate and permeate pressures were recorded. The same procedure was repeated for 4, 6, and 8 bar. Water hydraulic permeability (Lp) was determined from the slope of the linear association between normalized permeate flux and transmembrane pressure obtained at different operating pressures.

After the permeability test, the feed water (ultrafiltered water from the Lago Norte WTP spiked with E2 and BPA) was added to the feed reservoir and the experiment was initiated. Nanofiltration experiments were conducted for 12 h under operating pressures of 8 bar while monitoring pressure, flow rate, and temperature parameters. After each experiment, alkaline chemical cleaning with 0.05% NaOH solution was performed according to manufacturer’s recommendations [31], followed by a new permeability test to evaluate membrane cleaning efficiency and possible fouling effects.

Samples were collected throughout experiments, directly in amber glass bottles, and were kept on the fridge till analysis was performed.

BPA and E2 concentrations were determined by liquid chromatography (Agilent 1200 Series, Agilent Technologies, Palo Alto, CA, USA) coupled to mass spectrometry (3200 QTRAP, Sciex, Toronto, ON, Canada), using a LiChrosorb RP18 column (4.6 mm × 100 mm; 5 µm—Merck, Darmstadt, Germany). The mobile phase was methanol (A) and ultrapure water with 0.15% NH_4_OH (B) at a constant flow rate of 0.35 mL/min and temperature of 40 °C. Gradient elution was achieved by decreasing B from 50% (initial condition) to 30% in 0.2 min, to 15% in 1 min, held constant for 7 min and returning to the initial condition in 8 min. Under these conditions, BPA and E2 were eluted from the column at 7.7 and 9.8 min, respectively.

Prior to BPA and E2 quantification in each sample of feed water and permeate set, a new analytical curve was prepared. Standard solutions containing both BPA and E2 were prepared from the stock solutions in ultrapure water at concentrations of 5, 10, 20, 30, 40, 50, 60, and 70 µg/L.

Strict operational procedures were adopted throughout the analysis to minimize interference and ensure the reliability and reproducibility of the results. Once the samples were injected manually, at least three consecutive injections were performed for each sample, with additional injections being carried out whenever discrepancies between measurements were observed. To minimize contaminant accumulation and potential analytical interference, intermediate cleaning procedures were performed in the chromatographic system by injecting isopropanol after every three sample were analyzed.

The same rigor level was applied to establish and follow a cleaning protocol for glassware and materials used during contaminant handling and throughout the experiments, to avoid cross-contamination and external interference. The cleaning protocol was based on ASTM Method D7574 [32] for BPA analysis and EPA Method 539 [33] for hormone analysis.

As previously mentioned, the calibration range of the method was 5–70 µg/L, whereas the analyte concentration in the feed matrix ranged from 150 to 250 µg/L. Therefore, sample dilution was required prior to analysis. For this purpose, each sample was diluted with ultrapure water using dilution factors ranging from 2 to 7, based on the expected rejection performance of each membrane.

Each experiment was performed using a virgin membrane sheet, while its corresponding replicate was carried out using the same membrane sheet after chemical cleaning.

## 3. Results and Discussion

### 3.1. Calibration Parameters (LOD, LOQ, and Calibration Coefficients)

**Figure 2:**
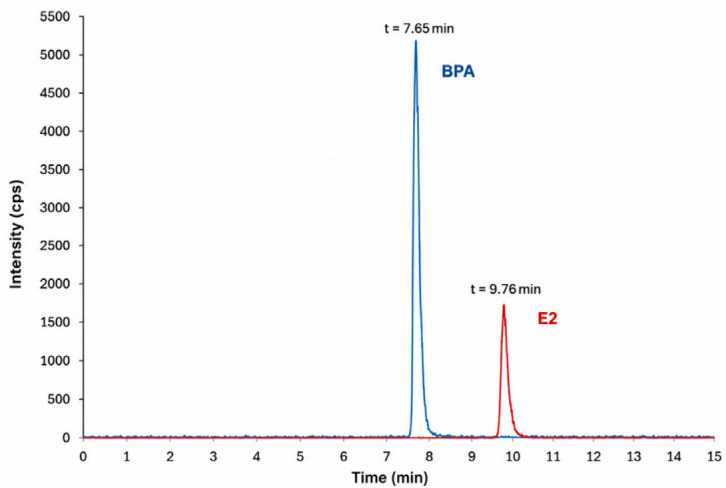
Chromatogram of the implemented analytical method.

Figure 2 shows a representative chromatogram of the implemented analytical method for E2 and BPA analysis.

As shown in the chromatogram, satisfactory separation between the two target contaminants was achieved. BPA eluted at approximately 7.65 min, whereas E2 eluted at 9.76 min. In addition, BPA produced a more intense signal than E2 at the same concentration, indicating a higher instrumental response under the selected analytical conditions.

The analytical performance characteristics of the method are summarized in Table 4, which presents the limits of detection (LODs) and quantification (LOQs), determined according to Eurachem’s guidelines [34].

As shown in Table 4, the limits of detection (LODs) for BPA and E2 were 1.4 and 0.58 µg/L, respectively, whereas the corresponding limits of quantification (LOQs) were 4.66 and 1.93 µg/L.

**Figure 3:**
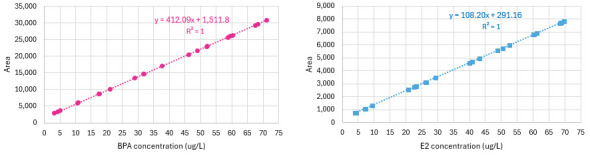
Working range and linearity of the analytical method.

The working range and linearity of the analytical method were evaluated by constructing calibration curves relating contaminant concentration to the corresponding chromatographic peak area. Figure 3 presents the linear regression results for the BPA and E2 datasets.

The linear regression coefficients were very high for both contaminants, demonstrating excellent linearity and confirming that the implemented analytical method can be reliably applied over the concentration range from the limit of quantification (LOQ) to 70 µg/L. Accordingly, the working range of the method was established as 5–70 µg/L for both BPA and E2.

### 3.2. Hydraulic Performance

As displayed in the Supplementary Materials (Table S1), a total of 16 experiments were carried out in the nanofiltration system; eight with the NFM1 membrane, and four experiments for NFM2 and NFM3 each.

The feed flow rate was kept constant throughout all experiments and reached a value of 2.21 ± 0.27 L/min. Since the experiments were performed using a SEPA CF Cell nanofiltration unit, the cross-sectional area of the flow channel was 0.000073 m^2^. Based on these values, the calculated crossflow velocity was 0.50 m/s.

Table 5 shows that, despite fluctuations in the measured values, the variation in transmembrane pressure (TMP) for the three membranes used was not significative. This behavior was expected due to the high quality of the ultrafiltered water and because all experiments were performed under the same operating pressure conditions. The TMP behavior during experiments with the selected membranes can be observed in Figures S4–S6 presented in the Supplementary Materials.

The hydraulic performance of the evaluated membranes differed significantly. NFM1 presented the highest pure water hydraulic permeability values, ranging from 19.59 to 22.74 L/h·m^2^·bar depending on the membrane sheet used. In contrast, NFM3 showed intermediate permeability values, between 11.16 and 12.22 L/h·m^2^·bar, while NFM2 exhibited the lowest permeability among the three membranes, ranging from 6.68 to 8.93 L/h·m^2^·bar. Although some studies have reported similar molecular weight cut-off (MWCO) values for NFM1 and NFM2 [22], the permeability results suggest structural differences between these membranes. Such behavior reinforces that membrane performance cannot be explained exclusively by MWCO or average pore size, since factors such as active layer thickness, pore size distribution, surface roughness, hydrophobicity, membrane compaction, and solute–membrane interactions also strongly influence hydraulic resistance and water transport.

**Figure 4:**
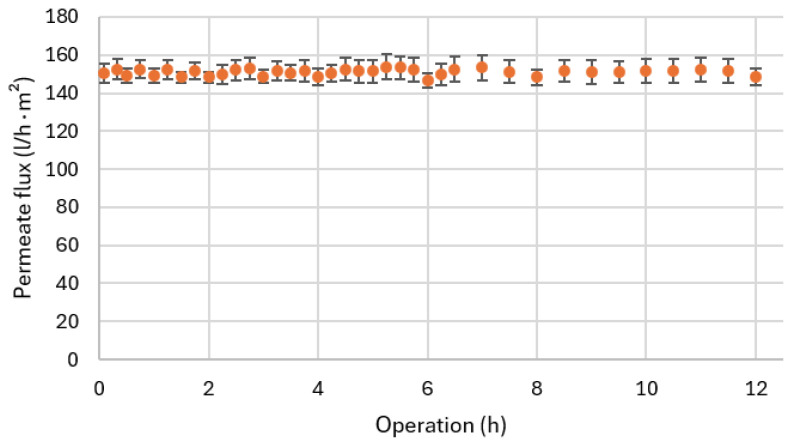
Permeate flux, normalized to 25 °C, during all experiments with the NFM1 membrane (n = 8).

**Figure 5:**
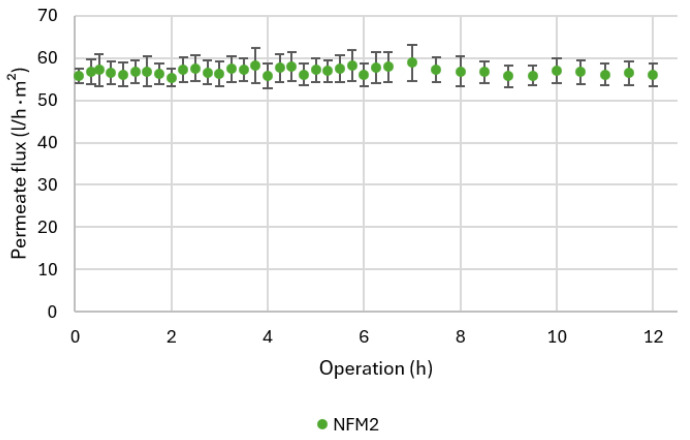
Permeate flux, normalized to 25 °C, during all experiments with the NFM2 membrane (n = 4).

**Figure 6:**
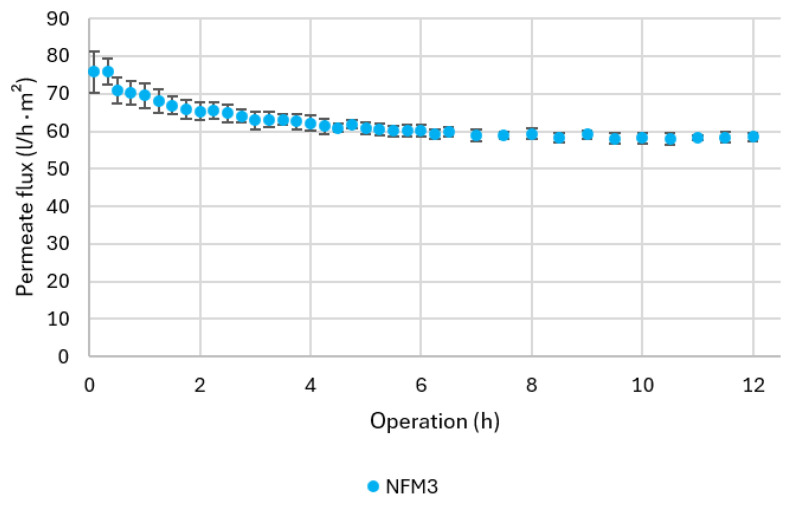
Permeate flux, normalized to 25 °C, during all experiments with the NFM3 membrane (n = 4).

As shown in Figure 4, Figure 5 and Figure 6, the permeate flux behavior followed the same trend observed for hydraulic permeability. NFM1 exhibited the highest permeate fluxes, ranging from 136.1 ± 2.8 to 171.7 ± 2.8 L/h·m^2^ due to its more open structure, characterized by larger effective pore diameter and higher MWCO. NFM3 showed intermediate permeate fluxes between 59.1 ± 2.9 and 67.0 ± 6.1 L/h·m^2^. Lastly, NFM2 presented the lowest permeate fluxes, ranging from 51.2 ± 0.5 to 63.3 ± 2.1 L/h·m^2^.

Different from what was observed for the NFM1 and NFM2 membranes, for the NFM3 membrane, permeate flux decreased over time, reaching a more stable performance after some hours, which indicates the occurrence of concentration polarization phenomena. Then, it was possible to apply the Hermia model, adapted to crossflow operation [35], to identify the mechanism that best explains the fouling behavior.

As shown in Figure 4 and Figure 5, the permeate flux remained nearly constant throughout the entire filtration period for the experiments performed with the NFM1 and NFM2 membranes. In contrast, as shown in Figure 6, a decline in permeate flux was observed during the first hours of operation in experiments with NFM3 membrane, which is indicative of membrane fouling. Therefore, the Hermia model was applied exclusively to the NFM3 membrane.

In the proposed model [36], a predominant fouling mechanism is identified based on the parameter “n”, which is associated with the different fouling mechanisms. In this context, “n” may assume the following values: n = 0 (cake layer), n = 1 (partial pore blocking), n = 1.5 (internal pore blocking), and n = 2 (complete pore blocking). By applying the model’s equations and plotting the permeate flux as a function of time, a linear association is obtained, in which the slope corresponds to the constant associated with each fouling mechanism. The predominant fouling mechanism is determined by evaluating the coefficient of determination (R^2^) for each linear fit. Therefore, the model presenting the R^2^ value closest to 1 is considered the mechanism that best explains the fouling behavior.

Considering the highest R^2^ values (Table 6), there appears to be a slight predominance of the cake layer formation mechanism, in which particles accumulate and deposit on the membrane surface without completely blocking the pores, forming a permeable and relatively uniform gel-like layer that acts as an additional filtration barrier [36,37]. This cake layer promotes solute accumulation near the membrane surface, intensifying osmotic pressure and contributing to permeate flux reduction [38].

Using the NFM1 membrane to treat biologically treated wastewater under different pH conditions, Ref. [38] observed, through the application of the Hermia model, that pH can influence the predominant fouling mechanism. At pH 6, complete pore blocking (n = 2) was identified as the dominant mechanism, whereas at pH values of 7 and 8, cake layer formation (n = 0) became predominant. Considering that the pH of the feed matrix used in the present study remained between 7 and 8 throughout the experiments, the predominance of cake layer formation (n = 0) observed in this work is consistent with the findings reported by [38].

### 3.3. Concentration in Permeate and Rejection

BPA and E2 concentrations in the permeate were monitored throughout all 16 experiments, as shown in Figures S1–S3. The concentration behavior varied slightly depending on the contaminant and the membrane. However, in general, the concentration showed an increase in the first two hours of operation, followed by less variable behavior.

A non-parametric statistical test (Kruskal–Wallis) was applied to evaluate whether significant differences existed in the permeate concentrations of E2 and BPA between the experiments carried out with the same membrane. As shown in more detail in Table S2 from the Supplementary Materials, the experiments conducted with the NFM1 membrane exhibited variability in permeate concentration, especially for BPA, as indicated by *p*-values lower than 0.05. A similar trend was also observed for the NFM2 and NFM3 membranes albeit to a lesser extent.

#### 3.3.1. E2 Rejection

**Figure 7:**
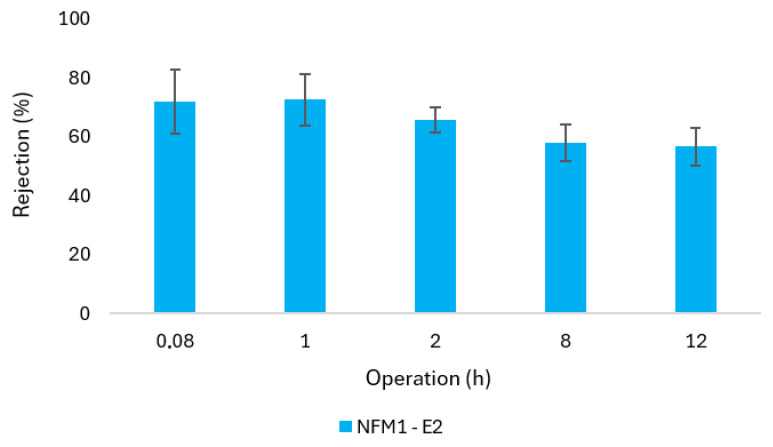
E2 rejection by NFM1 during all experiments (n = 8).

**Figure 8:**
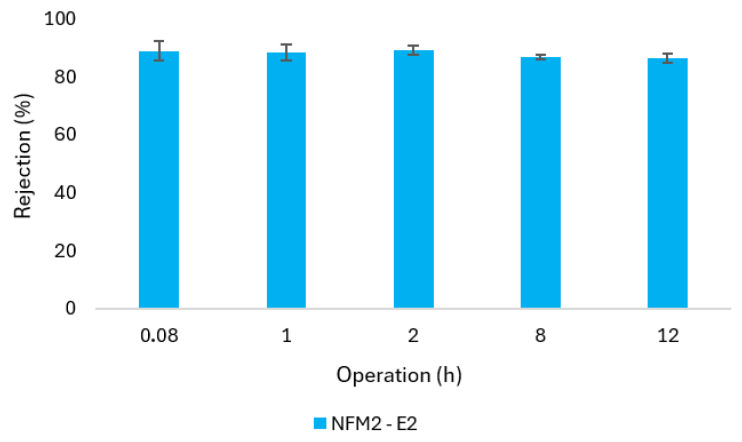
E2 rejection by NFM2 during all experiments (n = 4).

**Figure 9:**
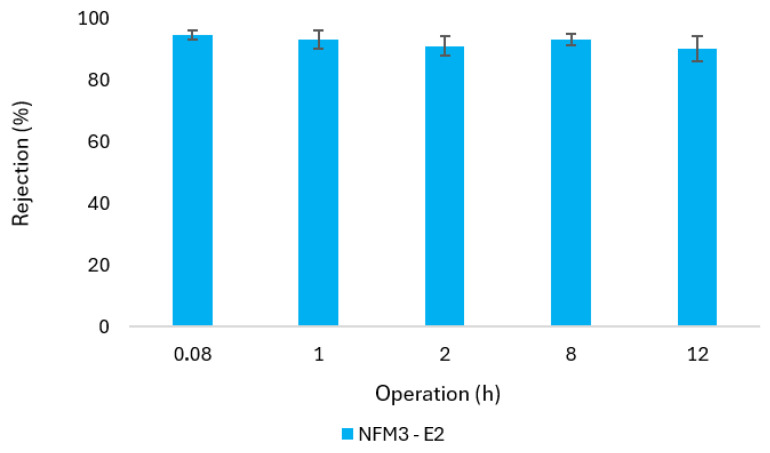
E2 rejection by NFM3 during all experiments (n = 4).

As displayed in the Supplementary Materials (Table S1), a total of 16 experiments were carried out in the nanofiltration system; eight with the NFM1 membrane and four experiments for NFM2 and NFM3 each. In this context, the E2 rejection obtained in all experiments, for each of the membranes, is presented in Figure 7, Figure 8 and Figure 9.

E2 rejection exhibited distinct behavior among the membranes evaluated under an operating pressure of 8 bar, mainly reflecting the structural and hydraulic differences in each membrane. Overall, NFM3 showed the highest rejection values, followed by NFM2 and NFM1. This behavior suggests the predominant influence of the size exclusion mechanism, since NFM3 is the most restrictive membrane among the three, presenting the lowest molecular weight cut-off (MWCO) and the smallest effective pore diameter. Also, no influence of electrostatic repulsion is expected since the pH of feed matrix ranged from 6.5 to 7.5, which is below the pKa values of E2 and BPA, as presented in Table 3. In contrast, NFM1, considered a loose membrane, showed the lowest rejection values, while NFM2 exhibited intermediate behavior, consistent with its partially similar structure to NFM1 but lower water hydraulic permeability.

From the error bars shown in Figure 7, Figure 8 and Figure 9, it is noticeable that the NFM2 and NFM3 membranes presented the lowest variability in E2 rejection, while the highest variability in rejection was observed with the NFM1 membrane.

For NFM1 membrane, E2 rejection ranged from 56 to 73%. Experiments with membrane sheets that presented lower hydraulic permeability and lower permeate fluxes generally produced higher rejection values, indicating that small structural differences between membrane sheets can affect separation performance. The rejection values obtained in this study were lower than those reported elsewhere, close to 80% and 90% [10,11], in which lower initial concentrations, around 100 ng/L, were used. On the other hand, one study, carried out with operating pressure of 6.9 bar and an E2 concentration of 100 µg/L in deionized water matrix, reported rejection of approximately 40% [13], which is similar to the lower limit obtained in the present study. These results demonstrate that E2 rejection by NFM1 depends on the combined effects of initial concentration, pressure, organic matter presence, and concentration polarization phenomena. Since the pH of the feed water in the experiments remained around 7 to 8, below the pKa of E2 (10.4), the hormone remained predominantly in its non-ionized form, reducing the influence of electrostatic repulsion and reinforcing the predominance of size exclusion.

The NFM2 membrane showed E2 rejection between 86 and 89% under 8 bar. Although some authors have reported similar MWCO values for NFM2 and NFM1 [22,23], the water hydraulic permeability and permeate flux results suggest that NFM2 has a more restrictive structure for water transport. This may explain the higher E2 rejection values observed for NFM2. Furthermore, compared to NFM1, E2 rejection by NFM2 showed lower sensitivity to variations in E2 concentration in feed water. The results indicate that, although size exclusion is the main removal mechanism, additional structural characteristics of the active layer, such as effective thickness, compaction, and surface roughness, also play important roles in contaminant transport.

The NFM3 membrane presented the highest E2 rejection values under 8 bar, ranging from 90 to 95%, as well as less variable behavior over time. The results obtained were consistent with those reported in the literature for this membrane of 90% and 99% with initial E2 concentrations of 100 ng/L and 100 µg/L, respectively [10,13]. Comparisons between studies indicates that NFM3 maintains high removal efficiency under different experimental conditions, suggesting a lower influence of initial concentration on rejection. In addition to size exclusion, the higher hydrophobicity of NFM3 may have favored hydrophobic interactions and adsorption phenomena between E2 and the membrane surface, especially during the initial stages of operation. Since E2 remained predominantly non-ionized at the experimental pH, the contribution of electrostatic repulsion was limited.

#### 3.3.2. BPA Rejection

**Figure 10:**
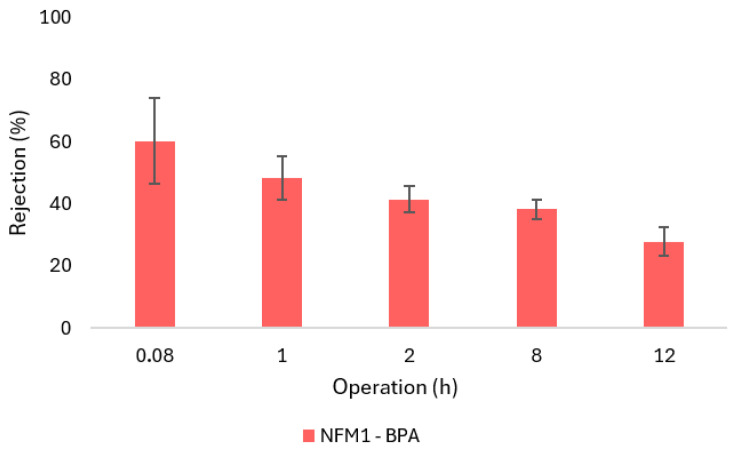
BPA rejection by NFM1 during all experiments (n = 8).

**Figure 11:**
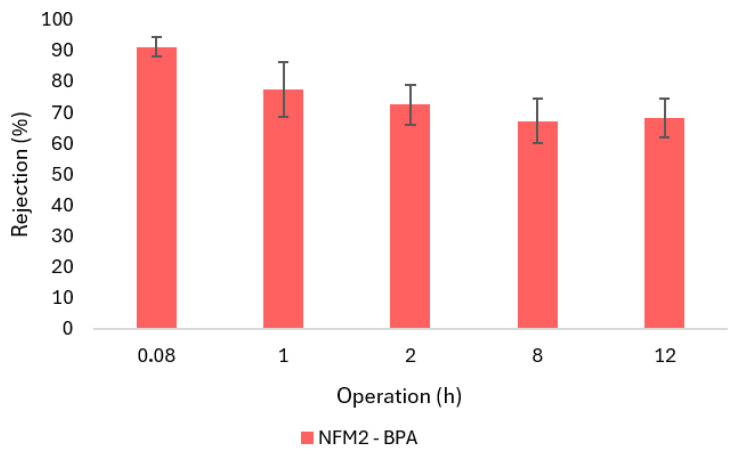
BPA rejection by NFM2 during all experiments (n = 4).

**Figure 12:**
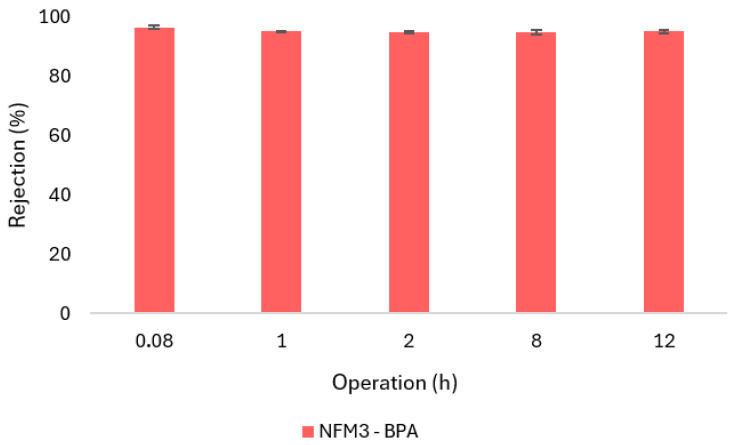
BPA rejection by NFM3 during all experiments (n = 4).

As presented in Figure 10, Figure 11 and Figure 12, the behavior of BPA rejection followed a trend similar to that observed for E2 albeit with generally lower rejection values, especially for NFM1. This occurs because BPA has a lower molecular weight than E2—that is, 228 g/mol and 272 g/mol, respectively—favoring its passage through nanofiltration membranes. Overall, the same performance order was observed as NFM3 > NFM2 > NFM1, and the variability in BPA rejection by the NF membranes also followed this same order, as shown in Figure 7, Figure 8 and Figure 9.

NFM1 showed the lowest BPA rejection values, ranging from 28 to 60%, in addition to greater variability throughout operation. The results obtained in this study were consistent with previous studies reporting BPA rejection of around 45% using deionized water and BPA concentration of 750 µg/L [18]. Other authors have reported rejection values close to 65% under 8.3 bar [11], while lower rejection values, around 20%, were observed under 6.9 bar and a BPA concentration of 100 µg/L [13]. Comparison among studies indicates that initial concentration alone does not explain rejection behavior, suggesting a strong influence of feed matrix characteristics and hydrodynamic conditions. The presence of organic matter, for example, may increase rejection by forming an additional layer over the membrane surface, thereby reducing the effective pore size. Previous studies observed that the addition of humic acid increased BPA rejection to approximately 60–70% [11,18]. Since the pKa of BPA ranges from 9.6 to 10.2, the compound remained predominantly non-ionized at the pH adopted in this study, indicating the predominance of size exclusion and adsorption mechanisms.

The NFM2 membrane exhibited intermediate BPA rejection, ranging from 67 to 91% in experiments conducted at 8 bar, which was higher than that observed for the NFM1 membrane. However, BPA rejection by NFM1 appears to be more sensitive to variations in concentration in the feed water than E2 rejection. The results highlight the more restrictive character of NFM2 compared to NFM1. The results also suggest that structural characteristics of the active layer, in addition to MWCO, significantly influence the removal of organic compounds.

The NFM3 membrane presented the highest BPA rejection values under 8 bar, ranging from 95 to 97%, in addition to less variable behavior over time. These results were higher than those reported elsewhere, which reported rejection close to 80% using BPA concentrations of 50 mg/L under 10 bar [22], probably due to very high concentration and higher operation pressure. The high rejection achieved by the NFM3 membrane emphasizes the predominance of size exclusion, favored by its lower MWCO and smaller pore diameter. In addition, the greater hydrophobicity of NFM3 may have intensified hydrophobic interactions and adsorption phenomena between BPA and the membrane surface. Previous studies have discussed that adsorption plays an important role in the removal of hydrophobic organic compounds by nanofiltration membranes [10,13,19]. Therefore, the results obtained indicate that the high BPA rejection achieved by NFM3 results from the combined effects of size exclusion and hydrophobic solute–membrane interactions.

## 4. Summary and Conclusions

The present work evaluated, at the bench-scale, the rejection of BPA and E2 by three different commercial nanofiltration membranes and their operational performance under operating pressure of 8 bar.

It is worth emphasizing that the contribution of this study lies in the simultaneous evaluation of BPA and E2 in the same matrix and in the use of a real water matrix-ultrafiltered water collected from a drinking water treatment plant (Lago Norte WTP). Furthermore, the findings are directly relevant to the local community, as they demonstrate the potential application of nanofiltration as a post-treatment process for a DWTP that supplies drinking water to several districts of the city of Brasília.

Based on the results presented, it can be concluded that:

• The NFM1 membrane showed better hydraulic performance, producing permeate fluxes two to three times higher than those produced by the other two membranes, NFM2 and NFM3.
• NFM3 displayed higher rejections between 90 and 95% for E2 and between 95 and 97% for BPA. Furthermore, it showed a more stable behavior throughout system operation. NFM2 exhibited intermediate behavior, with E2 rejection ranging from 86 to 89% and BPA rejection from 67 to 91%. Lastly, the NFM1 membrane showed the poorest rejection performance, with E2 rejection between 57 and 73% and BPA rejection between 28 and 60%.
• Size exclusion was the predominant mechanism of rejection by the three evaluated membranes. However, the adsorption of contaminants onto the membrane surface also contributed to rejection during the initial hours of operation, especially for the loose membranes, such as NFM2 and NFM1.
• A concentration polarization phenomenon was evident for the NFM3 membrane. However, this phenomenon did not appear to influence contaminant rejection.

The results confirm a trade-off between permeate production and contaminant rejection, with no single membrane outperforming all others across all criteria. The NFM3 membrane would be a safe option to efficiently reject BPA, E2, and other micropollutants at the Lago Norte WTP. On the hand, it is important to investigate other membranes and operating conditions to identify a combination capable of achieving high contaminant rejection combined with improved hydraulic performance.

## Figures and Tables

**Table 1:** Characterization of the feed matrix.

Physicochemical Characteristics	Results
pH	6.5–7.5
Alkalinity (mg/L CaCO_3_)	18.0–31.0
Conductivity (µS/cm)	28.1–99.8
Turbidity (NTU)	0.27–0.44
Absorbance	0.004–0.008
Hardness	13.0–22.0
Magnesium (mg/L)	1.0–2.9
Calcium (mg/L)	3.6–4.8
Temperature (°C)	25.0

**Table 2:** Physicochemical characteristics of BPA and E2.

Physicochemical Characteristics	BPA	E2
Molecular formula	C_15_H_16_O_2_	C_18_H_24_O_2_
Molar mass (g/mol)	228 ^a^	272 ^c^
pKa	10.2 ^a^	10.4 ^d^
Log Kow	3.32 ^b^	4.01 ^c^
Water solubility (mg/L)	129 ^b^	13 ^e^

^a^ [16]; ^b^ [18]; ^c^ [19]; ^d^ [20]; ^e^ [21].

**Table 3:** Characteristics of the selected membranes.

Parameter	NFM1	NFM2	NFM3
Membrane active layer	Semi-aromatic polypiperazine-amide ^a^	Semi-aromatic polypiperazine-amide ^a^	Fully aromatic polyamide ^a^
MWCO (Da)	200–300 ^b^; 300–400 ^c^; 400 ^d^	200–300 ^a^	200 ^b^
Pore diameter (nm)	0.76 ^c^; 0.84 ^e^; 0.87 ^f^	0.80 ^f^	0.62 ^c^; 0.68 ^g^
Contact angle (°)	23.4 ^g^	28 ^h^	42.2 ^g^
Water permeability (L/m^2^.h.bar)	13.5 ^g^	6.1 ^i^	6.4 ^g^
pH operating range	3–10 ^a^	2–12 ^a^	2–11 ^a^
Maximum operating pressure (bar)	41 ^a^	41 ^a^	41 ^a^
Maximum operating temperature (°C)	45 ^a^	45 ^a^	45 ^a^
Manufacturer	M1	M2	M1

^a^ from the manufacturer; ^b^ [22]; ^c^ [23]; ^d^ [24]; ^e^ [25]; ^f^ [26]; ^g^ [18]; ^h^ [27]; ^i^ [28].

**Table 4:** Detection and quantification limits of BPA and E2.

Contaminant	LOD (µg/L)	LOQ (µg/L)
BPA	1.4	4.7
E2	0.6	1.9

**Table 5:** Transmembrane pressure during all experiments with membranes NFM1, NFM2, and NFM3.

Pressure (bar)	Minimum	Maximum	Mean	Standard Deviation
NFM1	7.2	8.3	7.8	0.2
NFM2	7.4	8.4	7.9	0.2
NFM3	7.2	8.1	7.7	0.2

**Table 6:** The coefficient of determination (R^2^) obtained from the application of the Hermia model to experiments E7 and E8.

Experiment	Cake Layer (n = 0)	Partial Pore Blocking (n = 1)	Internal Pore Blocking (n = 1.5)	Complete Pore Blocking (n = 2)
E7	0.864	0.8334	0.816	0.7974
E8	0.9025	0.883	0.8719	0.8598

## Data Availability

Data are available from the authors upon reasonable request.

## Supplementary Materials

The following supporting information can be downloaded at: https://www.mdpi.com/article/10.3390/membranes16070242/s1, Table S1: Number of experimental runs and operating conditions employed; Figure S1: Permeate and feed concentrations during all experiments with the NFM1 membrane (n = 8); Table S2: Statistical analysis (non-parametric method: Kruskal–Wallis) of E2 and BPA concentrations in the permeate during experiments with the NFM1 membrane; Figure S2: Permeate and feed concentrations during all experiments with the NFM2 membrane; Table S3: Statistical analysis (non-parametric method: Kruskal–Wallis) of E2 and BPA concentrations in the permeate during experiments with the NFM2 membrane; Figure S3: Permeate and feed concentrations during all experiments with the NFM3 membrane; Table S4: Statistical analysis (non-parametric method: Kruskal–Wallis) of E2 and BPA concentrations in the permeate during experiments with the NFM3 membrane; Figure S4: TMP during operation of all experiments with NFM1 membrane; Figure S5: TMP during operation of all experiments with NFM2 membrane; Figure S6: TMP during operation of all experiments with NFM3 membrane.
