# Supplementary material for: Bench-Scale Evaluation of Hydraulic Performance and Rejection of Bisphenol-A and Estradiol by Different Nanofiltration Membranes as a Post-Treatment Step at the Lago Norte WTP—Brasília/DF, Brazil
Source: Membranes (Basel). 2026 Jul 17;16(7):242. doi: 10.3390/membranes16070242 (PMC13413878; doi:10.3390/membranes16070242)
Supplement: Supplementary file 1 [file membranes-16-00242-s001.zip › membranes-4406547-supplementary.pdf]

Supplementary Material

Bench scale evaluation of operational performance and rejection of bisphenol-a and estradiol by different nanofiltration membranes as a post-treatment step at the Lago Norte WTP – Brasília/DF, Brazil.

Bianca Campos Gonçalves; Cristina Celia Silveira Brandão*; Sara Regina Morais Kollar

Environmental Technology and Water Resources Post-Graduation Program, Department of Civil and Environmental Engineering, University of Brasília (UnB), Brasília 70910-900, Brazil
231119312@aluno.unb.br (B.C.G.)
* Correspondence: cbrandao@unb.br

Table S1. Number of experimental runs and operating conditions employed

Experiment	Abbreviation	Membrane	Operating pressure
Experiment 1	E1	NFM1	8 bar
Replicate 1	R1		
Experiment 2	$E2_{BPA}$		
Replicate 2	$E2_{E2}$		
Experiment 3	E3		
Replicate 3	R3		
Experiment 4	E4		
Replicate 4	R4		
Experiment 5	E5	NFM2	
Replicate 5	R5		
Experiment 6	E6		
Replicate 6	R6		
Experiment 7	$E8_{BPA}$	NFM3	
Replicate 7	R8		
Experiment 8	$E9_{E2}$		
Replicate 8	R9		

UFW: Ultrafiltrated water from Lago Norte WTP

Comment: Each membrane sheet was used in two consecutive experiments: the original experiment (Ei) and its corresponding replicate (Ri), in which the same operational conditions as Ei were adopted.

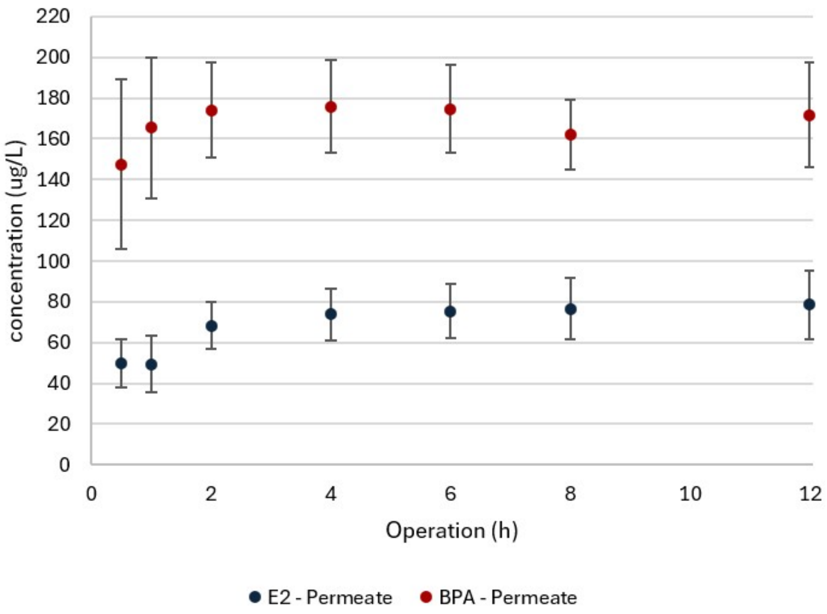

(a)

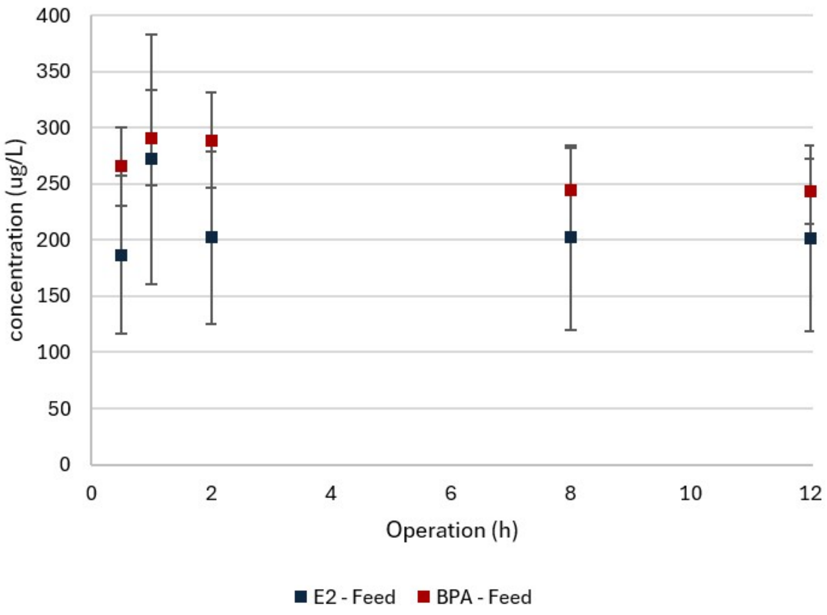

(b)

Figure S1. Permeate (a) and Feed (b) concentration during all experiments with NFM1 membrane (n=8).

For the statistical analysis, two hypotheses were defined: the null hypothesis (H_0) and the alternative hypothesis (H_1). The null hypothesis (H_0) assumed that the rejection values obtained in experiments Ei/Ri were equal to those obtained in experiments Ej/Rj, whereas the alternative hypothesis (H_1) assumed that the rejection values were different. If the p-value calculated by the statistical test was lower than the significance level (α = 0.05), the null hypothesis was rejected, indicating a statistically significant difference between the groups.

Table S2. Statistical analysis (non-parametric method: Kruskal-Wallis) of E2 and BPA concentration in the permeate during experiments with NFM1 membrane.

Experiments	P value		Comment
	E2	BPA	
All 8 experiments with NFM1 membrane	< 0,001	< 0,001	
E1 x R1	0,353	0,118	p > 0,05: E2 concentrations in permeate are statistically equal (E1 x R1; E1 x $E2_{E2}$; E1 x E3; E1 x R3; E1 x E4; E1 x R4; R1 x $E2_{E2}$; R1 x R3; R1 x E4; R1 x R4; $E2_{E2}$ x E4; $E2_{E2}$ x R4; E3 x R3; R3 x E4)
E1 x $E2_{E2}$ /$E2_{BPA}$	0,721	0,978	
E1 x E3	0,669	0,020	
E1 x R3	0,808	0,020	
E1 x E4	0,984	0,020	
E1 x R4	0,865	0,020	
R1 x $E2_{E2}$ /$E2_{BPA}$	0,862	0,127	
R1 x E3	0,020	1,000	
R1 x R3	0,080	0,944	
R1 x E4	0,670	0,108	
R1 x R4	0,865	0,020	
$E2_{E2}$ /$E2_{BPA}$ x E3	0,015	0,015	p > 0,05: BPA concentrations in permeate are statistically equal (E1 x R1; E1 x $E2_{BPA}$; R1 x $E2_{BPA}$; R1 x E3; R1 x R3; R1 x E4; E3 x R3; E3 x E4; E3 x E4)
$E2_{E2}$ /$E2_{BPA}$ x R3	0,021	0,015	
$E2_{E2}$ /$E2_{BPA}$ x E4	0,128	0,015	
$E2_{E2}$ /$E2_{BPA}$ x R4	1,000	0,015	
E3 x R3	0,683	0,992	
E3 x E4	0,029	0,427	
E3 x R4	0,029	0,029	
R3 x E4	0,120	0,276	
R3 x R4	0,020	0,029	
E4 x R4	0,029	0,029	

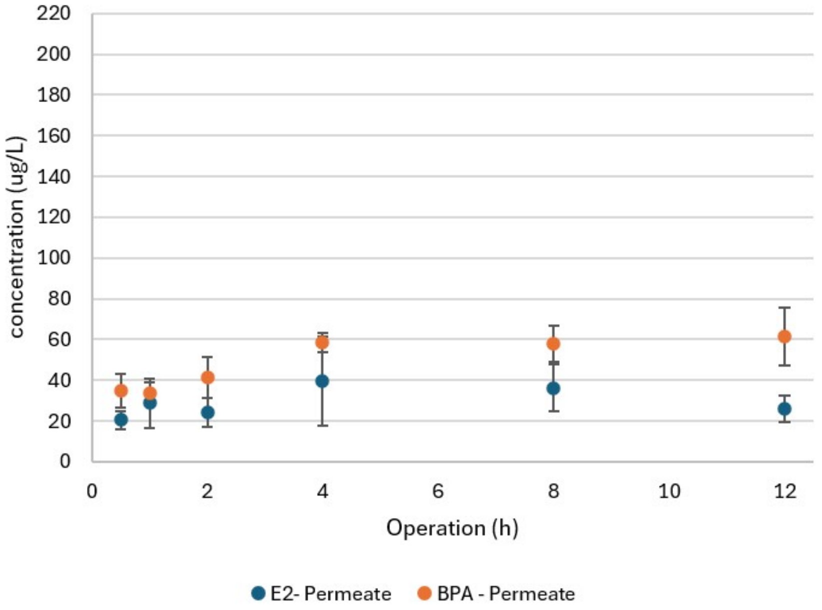

(a)

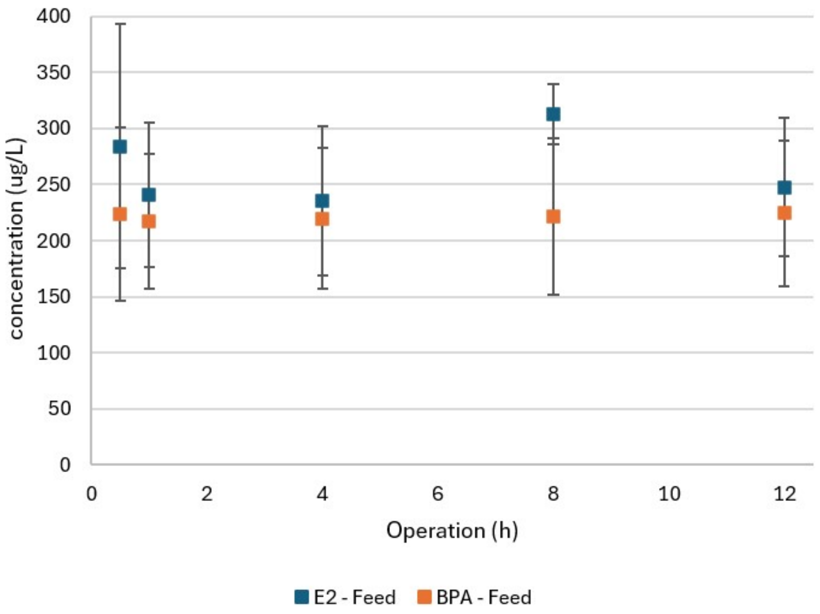

(b)

Figure S2. Permeate (a) and Feed (b) concentration during all experiments with NFM2 membrane.

Table S3. Statistical analysis (non-parametric method: Kruskal-Wallis) of E2 concentration in the permeate during experiments with NFM2 membrane.

Experiments	P value		Comment
	E2	BPA	
All 4 experiments with NFM2 membrane	< 0,001	< 0,001	p > 0,05: E2 concentrations in permeate are statistically equal (E5 x R5; E6 x R6)<br><br>p > 0,05: BPA concentrations in permeate are statistically equal (E5 x R5 x E6 x R6)
E5 x R5	0,961	0,232	
E5 x E6	0,028	0,024	
E5 x R6	0,028	0,012	
R5 x E6	0,024	0,036	
E5 x R6	0,024	0,017	
E6 x R6	0,656	0,995	

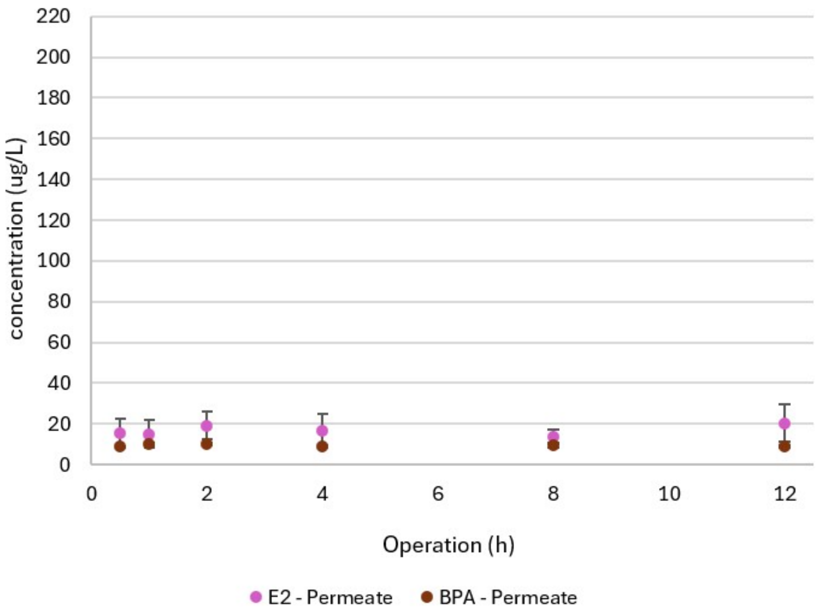

(a)

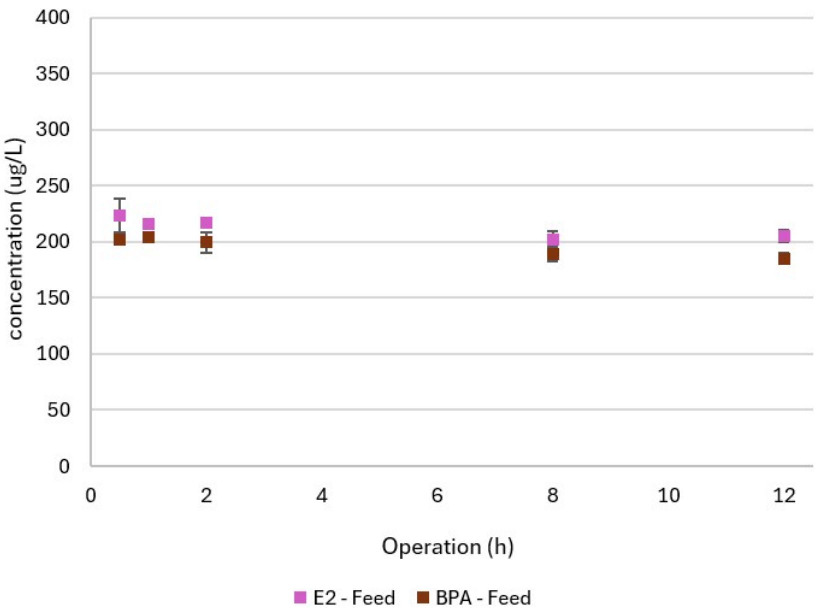

(a)

Figure S3. Permeate (a) and Feed (b) concentration during operation of all experiments with NFM3 membrane.

Table S4. Statistical analysis (non-parametric method: Kruskal-Wallis) of E2 and BPA concentrations in the permeate during experiments with NFM3 membrane.

Experiments	P value	Comment
E2		
All 4 experiments with NFM3 membrane	< 0,001	
$E9_{E2}$ x R9	0,899	
E9 x R8	0,002	

R9 x R8	0,008	p > 0,05: E2 concentrations in permeate are statistically equal (E9$_{E2}$ x R9)
BPA		
All 4 experiments with NFM3 membrane	0,002	p > 0,05: E2 concentrations in permeate are statistically equal (E8$_{BPA}$ x R8)
E8$_{BPA}$ x R8	0,270	
E8$_{BPA}$ x R9	0,034	
R9 x R8	0,002	

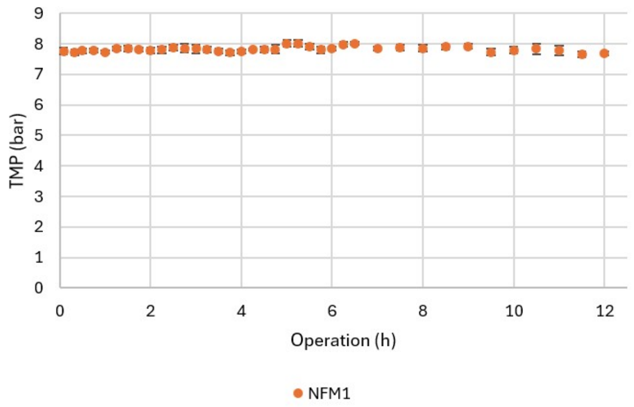

Figure S4. TMP during operation of all experiments with NFM1 membrane.

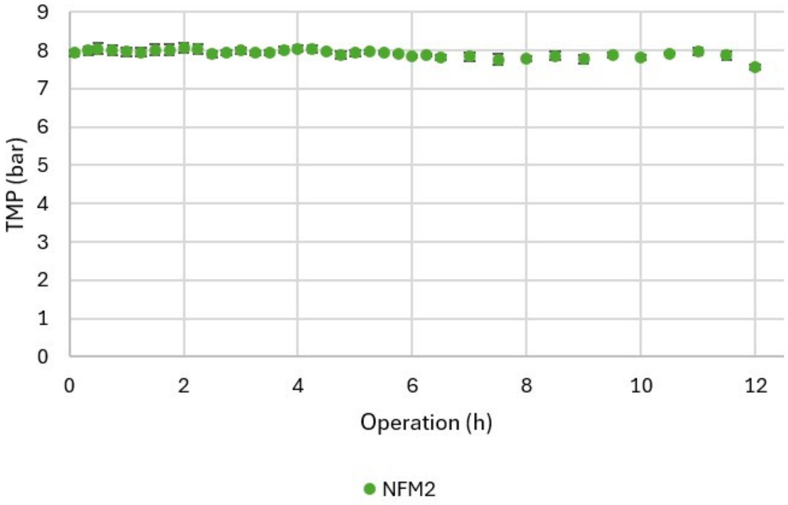

Figure S5. TMP during operation of all experiments with NFM2 membrane.

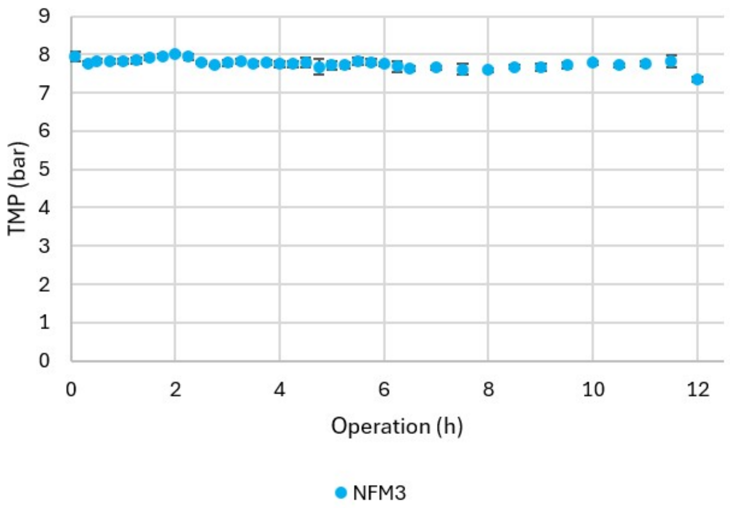

Figure S6. TMP during operation of all experiments with NFM3 membrane.
